# Electronic Monitoring Systems for Hand Hygiene: Systematic Review of Technology

**DOI:** 10.2196/27880

**Published:** 2021-11-24

**Authors:** Chaofan Wang, Weiwei Jiang, Kangning Yang, Difeng Yu, Joshua Newn, Zhanna Sarsenbayeva, Jorge Goncalves, Vassilis Kostakos

**Affiliations:** 1 School of Computing and Information Systems The University of Melbourne Carlton Australia

**Keywords:** hand hygiene, hand hygiene compliance, hand hygiene quality, electronic monitoring systems, systematic review, mobile phone

## Abstract

**Background:**

Hand hygiene is one of the most effective ways of preventing health care–associated infections and reducing their transmission. Owing to recent advances in sensing technologies, electronic hand hygiene monitoring systems have been integrated into the daily routines of health care workers to measure their hand hygiene compliance and quality.

**Objective:**

This review aims to summarize the latest technologies adopted in electronic hand hygiene monitoring systems and discuss the capabilities and limitations of these systems.

**Methods:**

A systematic search of PubMed, ACM Digital Library, and IEEE Xplore Digital Library was performed following the PRISMA (Preferred Reporting Items for Systematic Reviews and Meta-Analyses) guidelines. Studies were initially screened and assessed independently by the 2 authors, and disagreements between them were further summarized and resolved by discussion with the senior author.

**Results:**

In total, 1035 publications were retrieved by the search queries; of the 1035 papers, 89 (8.60%) fulfilled the eligibility criteria and were retained for review. In summary, 73 studies used electronic monitoring systems to monitor hand hygiene compliance, including application-assisted direct observation (5/73, 7%), camera-assisted observation (10/73, 14%), sensor-assisted observation (29/73, 40%), and real-time locating system (32/73, 44%). A total of 21 studies evaluated hand hygiene quality, consisting of compliance with the World Health Organization 6-step hand hygiene techniques (14/21, 67%) and surface coverage or illumination reduction of fluorescent substances (7/21, 33%).

**Conclusions:**

Electronic hand hygiene monitoring systems face issues of accuracy, data integration, privacy and confidentiality, usability, associated costs, and infrastructure improvements. Moreover, this review found that standardized measurement tools to evaluate system performance are lacking; thus, future research is needed to establish standardized metrics to measure system performance differences among electronic hand hygiene monitoring systems. Furthermore, with sensing technologies and algorithms continually advancing, more research is needed on their implementation to improve system performance and address other hand hygiene–related issues.

## Introduction

### Background

Hand hygiene is one of the most effective ways of reducing the transmission of pathogens that cause health care–associated infections (HAIs) [1-3]. HAIs are infections that people acquire in health care settings [4] and are the most crucial challenge to patient safety in health care [5]. HAIs dramatically increase patients’ length of stay, costs, mortality, and morbidity worldwide [6,7]. Moreover, HAIs also impose a heavy financial burden on health care systems. Solely in the United States, the estimated annual costs range from US $28 billion to US $45 billion [8]. The hands of health care workers (HCWs) represent the main pathway of pathogen transmission during health care [2], and Stone et al [9] estimated that at least one-third of HAIs can be prevented by achieving better hand hygiene in health care settings.

**Figure 1:**
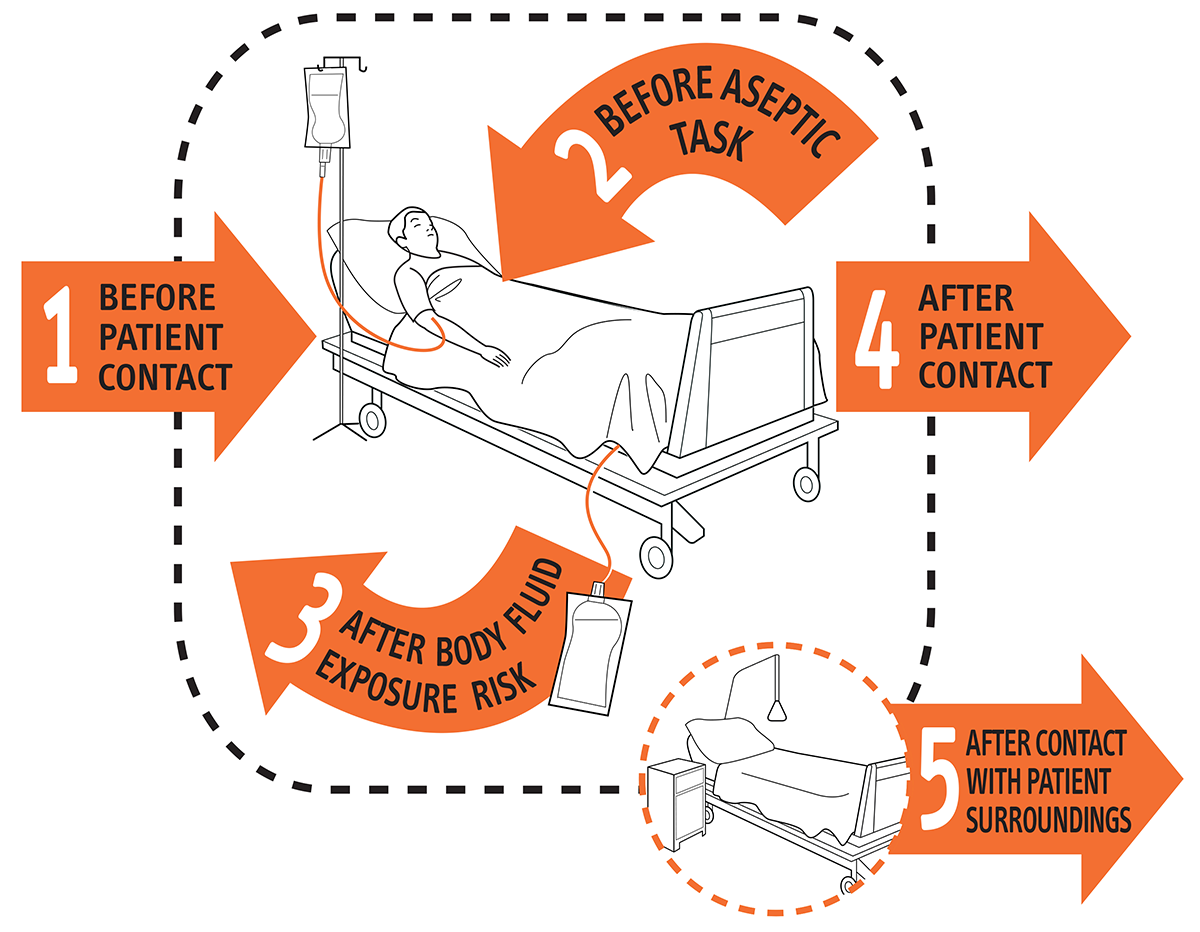
The key moments when health care workers should perform hand hygiene. Source: World Health Organization: “My 5 Moments for Hand Hygiene” (with permission) [2].

**Figure 2:**
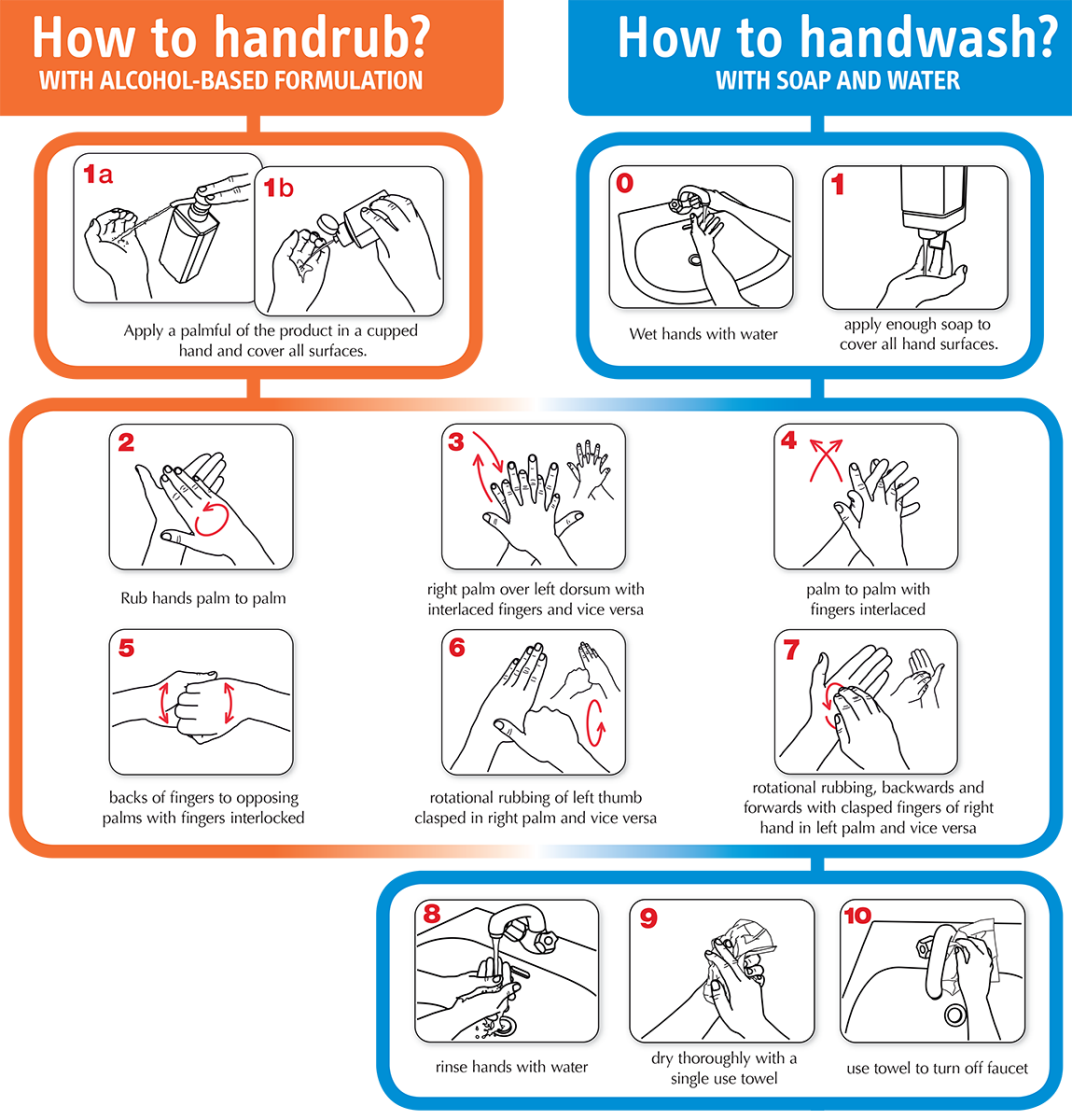
Standard World Health Organization procedures of alcohol-based hand rub and handwash with soap and water. Source: World Health Organization. How to Hand rub?/How to Handwash? (with permission) [10].

In 2009, the World Health Organization (WHO) issued the first *WHO guidelines on hand hygiene in health care* to provide a thorough review of evidence on hand hygiene in health care and specific recommendations to improve practices in health care settings [2]. In the guidelines, the WHO summarizes the five key moments when HCWs should ensure hand hygiene [2], as shown in Figure 1. The guidelines also recommend two standard hand hygiene techniques, *handwash with soap and water* for visibly soiled hands and *hand rub with alcohol-based formulation* for routine decontamination of hands [2], as shown in Figure 2.

However, research has found that hand hygiene compliance is often poor [11,12]. By summarizing 96 empirical studies, Erasmus et al [12] reported that the median compliance rate was only 40% among HCWs. Meanwhile, research also found that hand hygiene quality was unsatisfactory [13-15]. Szilágyi et al [15] reported that only 72% of HCWs could adequately clean all hand surfaces immediately after hand hygiene training. Owing to the importance of hand hygiene, these findings suggest that monitoring hand hygiene practices and providing HCWs with feedback regarding their performance are essential to promote hand hygiene compliance and quality in health care settings [16].

Direct observation by trained auditors is considered the gold standard for monitoring hand hygiene compliance in health care settings [2,17]. Self-reporting by HCWs and the measurement of hand hygiene product consumption are also widely used to monitor hand hygiene compliance [18]. However, Boyce et al [18,19] argued that the disadvantages of direct observation include time and resource consumption, insufficient sample size, lack of standardized observational practices, and the Hawthorne effect. Furthermore, self-reporting is not recommended by experts, as HCWs tend to overestimate their level of compliance, and the measurement of hand hygiene consumption cannot assess the appropriateness of HCWs’ hand hygiene timing and quality [18].

To assess hand hygiene quality, previous studies have used direct observation by trained auditors to observe HCWs’ compliance with the WHO 6-step hand hygiene technique [13,14,20]. Another common technique is using UV fluorescent substances to detect the surface coverage of hand hygiene products after hand hygiene [21,22]. Moreover, microbiological tests measure bacteria reduction count to evaluate hand hygiene quality [21,23,24]. However, using direct observation to monitor hand hygiene quality suffers from the same disadvantages as using direct observation to monitor hand hygiene compliance. Visual inspection of fluorescence is restricted to small sample sizes and a lack of standardized observational practices [25]. Furthermore, microbiological tests require time-consuming procedures and often overestimate the reduction of bacteria [21].

Given the above trade-offs, there has been increased interest in developing electronic monitoring systems to serve as an alternative or supplemental monitoring approach [19]. These electronic hand hygiene monitoring systems can be further categorized into electronic hand hygiene compliance monitoring systems and electronic hand hygiene quality monitoring systems.

Although previous reviews have described electronic hand hygiene compliance monitoring systems in detail, this is not the case for electronic hand hygiene quality monitoring systems [19,26,27]. Recent advances in sensor technologies and algorithms have also contributed to the development of new electronic hand hygiene monitoring systems. Furthermore, electronic hand hygiene monitoring systems have limitations that need to be identified and highlighted.

### Objectives

This paper aims to (1) review the literature regarding the latest technological developments in electronic hand hygiene systems for monitoring compliance and quality and (2) summarize the limitations and challenges when developing and deploying such systems in health care settings.

## Methods

### Search Strategy and Selection Criteria

We conducted a bibliographic search of the following web-based databases: PubMed, ACM Digital Library, and IEEE Xplore Digital Library. This systematic review followed the PRISMA (Preferred Reporting Items for Systematic Reviews and Meta-Analyses) [28] guidelines to reduce the risk of bias and increase its transparency and replicability. This systematic review is not registered on the network, and its review protocol is described below.

We derived the search query using a combination of key terms from previously published literature and expert advice. For the health-related database (PubMed), we specified search terms regarding hand hygiene, technological innovation, and observation to target electronic hand hygiene monitoring systems. For the technological databases (ACM Digital Library and IEEE Xplore Digital Library), we specified terms related to hand hygiene to include relevant technical innovations. The search queries for each database are given in Multimedia Appendix 1. Papers published between January 1, 2000, and June 30, 2020, were included in this study. As older literature is less relevant to today’s electronic hand hygiene monitoring systems, we decided to exclude it.

Studies were included if they (1) developed an electronic method or system to monitor hand hygiene compliance or hand hygiene quality, (2) used an existing electronic device or application to support hand hygiene monitoring, or (3) adopted an existing electronic hand hygiene monitoring system and provided sufficient technical details. Meanwhile, studies were excluded if they (1) did not explicitly target electronic hand hygiene monitoring, (2) did not provide adequate technical details (eg, communication protocol and sensor specification), (3) were not published in English, or (4) were not original research papers (eg, abstracts, review papers, and editorials).

To identify the relevant studies, we first imported the search results into a spreadsheet for duplicate removal. Then, the titles were screened based on the selection criteria. If a publication passed the title screening, its abstract was assessed. Finally, the decision for inclusion was made according to the full text of the study. A total of 2 authors, CW and WJ, independently performed the study selection procedure for the retrieved publications. Disagreements between the 2 authors were further summarized and resolved by discussion with the senior author, VK, whenever necessary.

### Data Extraction and Data Analysis

To collect information from the included studies in a consistent manner, we created a data extraction table (Multimedia Appendix 2). A total of 2 authors, CW and WJ, independently performed the data extraction procedure, whereas disagreements were resolved by discussion with the senior author, VK.

As we aimed to summarize the different technologies used in electronic hand hygiene monitoring systems, we adopted a narrative approach to synthesize the extracted data. All studies were first grouped by their study aims (monitoring either hand hygiene compliance or quality). After that, the categorized studies were further divided into several categories according to their technical details. Specifically, electronic hand hygiene compliance monitoring systems include (1) application-assisted direct observation, (2) camera-assisted observation, (3) sensor-assisted observation, and (4) real-time locating systems (RTLSs). Meanwhile, electronic hand hygiene quality monitoring systems include (1) measure compliance with the WHO 6-step hand hygiene techniques and (2) detect surface coverage or illumination reduction of fluorescent substances.

Owing to the high level of heterogeneity of the included studies, this study could not provide meta-analyses of the system performance and relevant HCWs’ behavior changes. The significant heterogeneity also resulted in missing standardized automation tools to evaluate the risk of bias and assess the certainty for each included study.

## Results

### Inclusion of Studies and Study Characteristics

**Figure 3:**
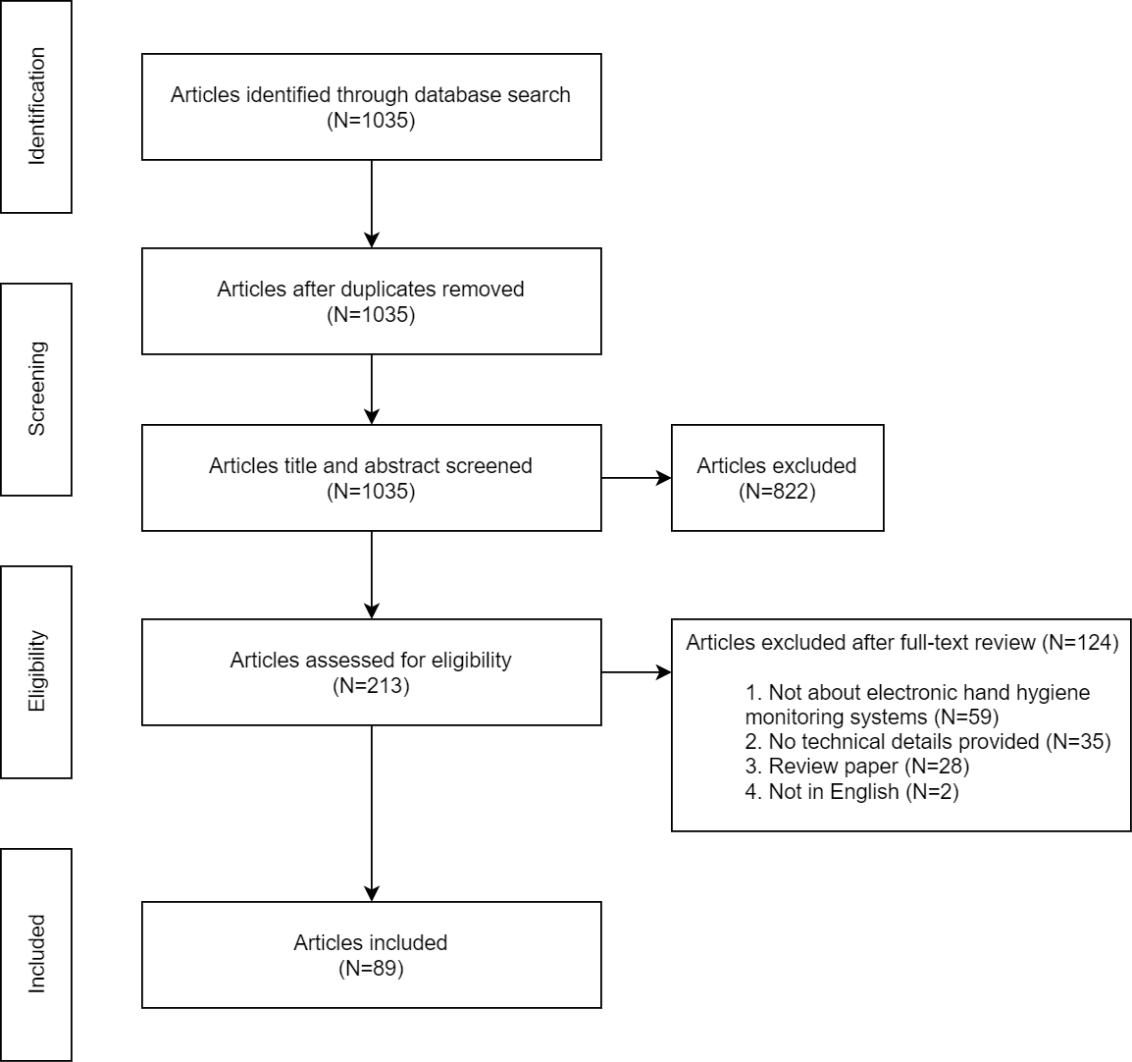
Flowchart of the selection process for the systematic review.

In total, 1035 publications were retrieved by the initial search queries (777/1035, 75.07% from PubMed; 190/1035, 18.36% from the IEEE Xplore Digital Library, and; 68/1035, 6.57% from the ACM Digital Library). None of the retrieved studies were removed based on duplication. After screening the titles and abstracts, 79.42% (822/1035) of studies were excluded for not meeting the eligibility criteria. Thus, 20.58% (213/1035) of studies were reviewed for the full text. Of these 213 studies, 124 (58.2%) studies were excluded. The main reasons for exclusion were the irrelevance of electronic hand hygiene monitoring systems (59/124, 47.6%) and insufficient technical details (35/124, 28.2%). No study was excluded if they met the inclusion criteria. Therefore, of the 213 studies, 89 (41.8%) fulfilled the eligibility criteria and were retained for review [25,29-116]. Figure 3 shows the process of searching for and selecting the studies included in the review.

All the 89 reviewed studies were published between 2009 and 2020, with 9 (10%) dated in or before 2010 [42,46,56,60,81,83,86,92,113], 38 (43%) dated between 2011 and 2015 [32,33,36,38,39,45,48,49,51,52,54,59,61,63-65,67, 68,72,73,78-80,82,84,87-89,93-97,99-101,108,116], and 42 (47%) dated in or after 2016 [25,29-31,34,35,37,40,41, 43,44,47,50,53,55,57,58,62,66,69-71,74-77,85,90,91, 98,102-107,109-112,114,115]. Regarding the countries where the studies were conducted, 6 countries had ≥5 studies: United States (31/89, 35%) [25,30,31,36,38,39,41,46-48,51,52,59, 61,63,68, 69,71,74,76,80-84,92,95,96,101,107,111], Canada (8/89, 9%) [42,72,86-89,98,100], Japan (7/89, 8%) [44,45,55,58,62,114,115], Brazil (6/89, 7%) [32,33,49,56,67,78], Germany (6/89, 7%) [37,40,50,66,108,109], and India (5/89, 6%) [64,65,94,99,102]. The demographic information of participants was provided in only 70% (62/89) of studies. Most studies (50/89, 56%) recruited HCWs from hospitals or clinics [29,30,32-40,44,46-57,59,61,66-68,73-75,77-80, 82,84,86,87,89-91,95,96,98-100,103,106], and few (2/89, 2%) studies also involved patients from hospitals [66,94]. The remaining studies recruited the general public (7/89, 8%) [45,60,64,65,109-111] or students (4/89, 4%) [76,104,114,115] from communities or educational settings.

### Compliance Monitoring Systems

We identified 73 studies that either implemented or adopted an electronic monitoring system for hand hygiene compliance and grouped them into 4 categories based on their enabling technology [19,26]: application-assisted direct observation (5/73, 7%), camera-assisted observation (10/73, 14%), sensor-assisted observation (29/73, 40%), and RTLS (32/73, 44%).

#### Application-Assisted Direct Observation

Approximately 7% (5/73) of studies used applications to assist trained auditors in observing hand hygiene compliance (details are included in Table 1) [29-33]. With these applications, human observers could record their observations using smartphones or tablets. Unlike manual observation with paper forms, application-assisted observation avoids the need for transcription, which could cause delays in analysis, increase the associated cost, and introduce errors [117]. In addition, the prevalence of smartphones and tablets in health care settings makes data collection more unobtrusive and reduces the Hawthorne effect [26]. Both in-house and commercial applications have been used for application-assisted direct observations.

The monitored hand hygiene opportunities may vary in different studies. Most studies followed the instructions given by the WHO *5 moments for hand hygiene* [29,31-33]. Conversely, Sickbert-Bennett et al [30] simplified the observation process to *patient room entry or exit* events (as proxies for moments 1, 4, and 5).

**Table 1:** Description of application-assisted direct observation studies.

Paper and system description		Required device	System type	System metrics (hand hygiene opportunities)
**Kariyawasam et al** **[29]**				
	Self-developed application	Android tablet	Research	WHO^a^ 5 moments
**Magnus et al** **[32] and Sodré da Costa et al** **[33]**				
	iScrub	iOS devices	Commercial	WHO 5 moments
**Sickbert-Bennett et al** **[30]**				
	iScrub	iOS devices	Commercial	Patient room entry/exit events
	SelectSurvey	Web browser	Commercial	Patient room entry/exit events
**Wiemken et al** **[31]**				
	Google forms	Web browser	Commercial	WHO 5 moments

^a^WHO: World Health Organization.

#### Camera-Assisted Observation

In contrast with application-assisted direct observation, which solely relies on human auditors, studies with camera-assisted observation could rely on either human auditors [34-40] or algorithms [41-43] for analysis (details included in Table 2). Approximately 30% (3/10) of studies installed cameras inside and outside patient rooms to capture all five hand hygiene moments [34-36]. Researchers manually coded the streaming and recorded videos. Armellino et al [38,39] recruited a remote video auditing company (Arrowsight, Inc) to conduct compliance observations only when HCWs entered or exited the patient room (as proxies for moments 1, 4, and 5). Rather than installing cameras in the environment, Diefenbacher et al [37,40] proposed mounting a camera on the chest of HCWs that aimed at their hands, and researchers further analyzed these first-person view video recordings according to the WHO 5 moments for hand hygiene.

In terms of automated analyses, Zhong et al [41] attached a red green blue (RGB) camera to the chest of HCWs to collect egocentric videos. By feeding RGB images and optical flow images inside a two-stream convolutional neural network, they identified hand hygiene events in HCWs’ daily routines [41]. Snoek et al [42] used an RGB camera with a microphone to observe handwash events in older adults with Alzheimer disease. Awwad et al [43] used an RGB-depth camera, Kinect (Microsoft Corporation), to achieve automatic detection of moment 1 (before touching a patient). Depth cameras generate pictures with stereo information, and these pictures have pixels with a value being the distance from the camera or *depth*. Hand hygiene compliance of moment 1 was then estimated by measuring the proximity between the subjects’ hands and patient/bed with the presence of hand rub events [43].

**Table 2:** Description of camera-assisted observation studies.

Paper and system description			Device location	Video type	System type	System metrics (hand hygiene opportunities)
**Auditor (human)**						
	**Brotfain et al** **[34]**					
		RGB^a^ camera	Patient room	Streaming	Research	WHO^b^ 5 moments
	**Sánchez-Carrillo et al** **[35]**					
		RGB camera	Patient room	Recorded	Research	WHO 5 moments
	**Diller et al** **[36]**					
		RGB camera (with infrared lens)	Patient room	Recorded	Research	WHO 5 moments
	**Armellino et al** **[38,39]**					
		RGB camera	Sink and sanitizer dispenser	Recorded	Commercial (Arrowsight)	Patient room entry/exit events
		Motion sensor	Patient room entrance	Recorded	Commercial (Arrowsight)	Patient room entry/exit events
	**Diefenbacher et al** **[37,40]**					
		RGB camera	HCWs^c^ (chest)	Recorded	Research	WHO 5 moments
**Auditor (algorithm)**						
	**Zhong et al** **[41]**					
		RGB camera	HCWs (chest)	Recorded	Research	Hand hygiene events
	**Snoek et al** **[42]**					
		RGB camera	Sink	Recorded	Research	Hand hygiene events (5 states related to faucet interaction)
		Microphone	Sink	Recorded	Research	Hand hygiene events (5 states related to faucet interaction)
	**Awwad et al** **[43]**					
		RGB depth camera	Patient bed	Recorded	Research	Moment 1 (before touching a patient)

^a^RGB: red green blue.

^b^WHO: World Health Organization.

^c^HCW: health care worker.

#### Sensor-Assisted Observation

Of the 73 studies, 29 (40%) observed hand hygiene compliance using sensors (details are included in Table 3) [32,33,44-70]. These studies were grouped into 3 categories on the basis of sensor type: electronic dispenser, electronic dispenser assisted by other sensors, and inertial measurement unit (IMU) and microphone.

Of these 29 studies, 15 (52%) used electronic dispensers to record the frequency of hand hygiene events and estimate the volume of hand hygiene products dispensed [32,33,44-50,54-57,59,67]. A range of sensors was used to trigger the electronic dispenser counter, including pressure resistors [45,53], magnetic sensors [66], and photosensors [58]. These records were then manually collected by researchers or wirelessly transmitted to the associated servers for further analysis. Compared with direct observation, electronic dispensers can capture hand hygiene events with substantially fewer personnel resources and are unaffected by the Hawthorne effect [19]. However, electronic dispensers cannot detect the hand hygiene opportunities specified by the WHO 5 moments for hand hygiene [19]. Thus, several studies supplemented hand hygiene events with further information to estimate hand hygiene compliance, including outpatient visit records, the expected number of hand hygiene events, ward-specific conversion factors, the number of patients in the unit, nurse visit records, and documented activities [44,48-50,55,57,59].

As electronic dispensers cannot detect hand hygiene opportunities according to the WHO guidelines, other sensors were used to capture these opportunities [51-53,61,62,66,68]. A common practice was to use motion sensors to record patient room entry or exit events (as proxies for moments 1, 4, and 5) [51,61,66]. Here, the dispensers and motion sensors uploaded the time stamp of dispense and room entry/exit events to a server. Once a motion sensor was activated, the server measured the occurrence of a hand hygiene event within a predefined period and thus estimated the hand hygiene compliance rate. Conversely, Geilleit et al [53] placed a motion sensor around HCWs’ working area and pressure plates on patient couches and chairs. Hand hygiene opportunities were defined as the movement of HCWs into a patient zone when the pressure plates were activated. Furthermore, studies used electronic dispensers with other sensors, including IMUs and microphones, to recognize different types of hand hygiene events from HCWs’ daily routines [52,62,68].

Of the 29 studies, 7 (24%) used an IMU and microphone to distinguish hand hygiene events from daily activities [58,60,63-65,69,70]. An IMU is an electronic sensor that measures a body’s specific force, angular rate, and orientation. Of the 7 studies, 2 (29%) attached an IMU wristband to users’ wrists to collect physical signals and utilize these signals to recognize hand hygiene events [63,69]. By using acceleration, gyration, and audio signals from participants’ wrists, Wijayasingha et al [70] applied the naive Bayes algorithm to identify both hand hygiene and oral hygiene events from people with developmental disabilities. Instead of placing sensors on users’ wrists, 43% (3/7) of studies embedded IMU sensors with or without microphones inside soap bars [60,64,65]. These augmented soap bars were then distributed to low-income households to monitor their soap use associated with hand and body wash. Furthermore, Miyazaki et al [58] attached a microphone to a sink to distinguish hand hygiene events from other daily activities.

**Table 3:** Description of sensor-assisted observation studies.

Paper and system description				Device location		System type		System metrics (hand hygiene opportunities)
**Electronic dispenser**								
	**Arai et al** **[44]**							
		Dispenser	Outpatient area		Commercial (Compleo-IO)		Outpatient visit records	
	**Asai et al** **[45]**							
		Dispenser	Hospital entrance		Research		Hand hygiene events	
	**Boyce et al** **[46]**							
		Dispenser	Patient room and hallway		Commercial (iSIGNOL)		Hand hygiene events	
	**Cohen et al** **[47]**							
		Dispenser	Throughout entire facility		Commercial (DebMed GMS)		Hand hygiene events	
	**Conway et al** **[48]**							
		Dispenser	Throughout entire facility		N/A^a^		Expected hand hygiene events	
	**Diefenbacher et al** **[50]**							
		Dispenser	Patient room		Commercial (Ingo-man Weco)		Hand hygiene events (conversion factor)	
	**Helder et al** **[54]**							
		Dispenser	Patient room		Commercial (ComSens NewCompliance)		Hand hygiene events	
	**Kato et al** **[55]**							
		Dispenser	Outpatient area		Commercial (CARECOM Co, Ltd)		Outpatient visit records	
	**Morgan et al** **[59]**							
		Dispenser	Patient room		N/A		Patients number	
	**De MacEdo et al** **[49]**							
		Dispenser	Patient room		Commercial (NXT 1-L model)		Nurse visits (nurse call system)	
	**Marra et al** **[56,67], Magnus et al** **[32], and Sodré da Costa et al** **[33]**							
		Dispenser	Patient room		Commercial (NXT 1-L model)		Hand hygiene events	
	**Scheithauer et al** **[57]**							
		Dispenser	Throughout entire facility		Commercial (Ingo-man Weco)		Documented activities	
**Electronic dispenser assisted by other sensors**								
	**Ellison et al** **[51]**							
		Dispenser	Throughout intensive care units		N/A		Patient room entry/exit events	
		Motion sensor	Patient room entrance		N/A		Patient room entry/exit events	
	**Sharma et al** **[61]**							
		Dispenser	Hallway		Research		Patient room entry/exit events	
		Motion sensor	Examination room entrance		Research		Patient room entry/exit events	
	**Gaube et al** **[66]**							
		Dispenser	Patient room and hallway		Research		Patient room entry/exit events	
		Motion sensor	Dispenser		Research		Patient room entry/exit events	
	**Geilleit et al** **[53]**							
		Dispenser	Examination room		Research		Patient room entry/exit events	
		Motion sensor	HCWs’^b^ work area		Research		Patient room entry/exit events	
		Pressure plate	Examination couch, Chair		Research		Patient room entry/exit events	
	**Galluzzi et al** **[52,68]**							
		Dispenser	N/A		Research		Hand hygiene events	
		IMU^c^	HCWs (wristwatch)		Research		Hand hygiene events	
	**Tobita et al** **[62]**							
		Dispenser	Sink		Research		Hand hygiene events	
		Microphone	Sink		Research		Hand hygiene events	
**IMU and microphone**								
	**Uddin et al** **[63]**							
		IMU	HCWs (wristband)		Research		Hand hygiene events	
	**Li et al** **[69]**							
		IMU	HCWs (wristband)		Research		Hand hygiene events	
	**Ram et al** **[60]**							
		IMU	Soap bar		Research		Hand hygiene events	
	**Wright et al** **[64] and Zillmer et al** **[65]**							
		IMU	Soap bar		Research		Hand hygiene events	
		Microphone	Soap bar		Research		Hand hygiene events	
	**Wijayasingha et al** **[70]**							
		IMU	HCWs (wristwatch)		Research		Hand hygiene events	
		Microphone	HCWs (wristwatch)		Research		Hand hygiene events	
	**Miyazaki et al** **[58]**							
		Microphone	Sink		Research		Hand hygiene events	

^a^N/A: not applicable.

^b^HCW: health care worker.

^c^IMU: inertial measurement unit.

#### Real-time Locating Systems

Of the 73 studies, 32 (44%) studies deployed RTLSs to track hand hygiene compliance (details included in Table 4) [67,71-101]. The RTLS was originally used to identify and track the location of objects or people in real time within a specified area. By sensing dispenser actuation and HCWs’ movements, servers from an RTLS can measure HCWs’ hand hygiene compliance rates as the ratio of dispenser actuation to the patient room or area entry or exit events (as proxies for moments 1, 4, and 5) [19]. On the basis of the underlying technology, we divided these systems into 6 categories: radio-frequency identification (RFID), infrared, ultrasound, Bluetooth low energy (BLE), IEEE 802.15.4/ZigBee, and Wi-Fi.

Of the 32 studies, 10 (31%) developed or deployed an RFID-based RTLS [71,73,75,76,83,84,90,93,97,99]. RFID uses radio waves to identify and track tags attached to objects. RFID tags can come in a variety of shapes and can be embedded into HCWs’ name tags, wristbands, bracelets, and even shoes. When HCWs with RFID tags pass RFID readers, the readers detect the HCWs’ tags and then communicate the collected information to a central server. To record HCWs’ hand hygiene events, 30% (3/10) of studies placed RFID readers either next to dispensers or embedded RFID readers into dispensers [71,76,93], and these RFID readers were activated by dispensing events. By installing RFID readers next to dispensers and at the entrance of patient rooms or around patient beds, RFID-based RTLS could recognize both hand hygiene events and the entry and exit of individuals into a patient room or a patient area [73,75,83,84,90,99]. Furthermore, several other sensors were used to assist in the observation of RFID-based RTLS, including motion sensors (recording movements around patient beds), IMUs (recording duration of hand hygiene events), and ethanol sensors (recognizing alcohol-based hand rub events) [90,97,99].

Of the 32 studies, 8 (25%) studies adopted infrared-based RTLSs to monitor hand hygiene compliance [72,74,77,86-89,98]. An infrared transmitter uses infrared light pulses to transmit a unique infrared code to its receiver, and the receiver can then estimate their relative position inside a building. For all 8 studies, infrared transmitters were installed across health care settings and continuously emitted their relative location information (eg, patient room, patient bed, and hallway). In addition, the transmitters were embedded in dispensers and activated for a short period after dispenser actuation. The infrared receivers were carried by HCWs and continuously received location information from the transmitter and counted HCWs’ hand hygiene events. In addition, 63% (5/8) of studies used a wearable dispenser to facilitate HCWs’ hand hygiene practices. Furthermore, an ethanol sensor was deployed in an infrared-based RTLS to recognize hand rub events rather than relying on a wall-mount dispenser [77].

Of the 32 studies, 2 (6%) studies deployed ultrasound-based RTLSs [79,100]. Similar to other RTLS, ultrasound-based RTLSs comprise transmitters, receivers, and dispensers. Transmitters emit sound in the ultrasonic range, and receivers detect these sounds and thus locate the transmitters. Unlike infrared-based RTLSs, ultrasonic transmitters were typically either placed in health care settings or carried by HCWs, and thus the sound contained either location information or HCWs’ identity. Through the collected signals, the receivers could locate the HCWs’ real-time location and recognize patient room entry/exit events. When dispensers were used in ultrasound-based RTLSs, transmitters or receivers were also embedded in these dispensers and transmitted dispensing events to the receivers.

**Table 4:** Description of real-time locating system studies.

Paper and system description				Device location		System type		System metrics (hand hygiene opportunities)
**Radio-frequency identification (RFID)**								
	**Decker et al** **[76]**							
		RFID tag	HCWs^a^ (tag)		Research		Class schedules	
		RFID reader	Dispenser		Research		Class schedules	
	**Bal et al** **[71]**							
		RFID tag	HCWs (tag)		Research		Hand hygiene events	
		RFID reader	Dispenser and faucet		Research		Hand hygiene events	
		Dispenser/faucet	Patient room entrance and patient bed		Research		Hand hygiene events	
	**Meydanci et al** **[93]**							
		RFID tag	HCWs (wristband)		Research		Hand hygiene events	
		RFID reader	Dispenser		Research		Hand hygiene events	
		Dispenser	Patient room and hallway		Research		Hand hygiene events	
	**Boudjema et al** **[73] and Brouqui et al** **[75]**							
		RFID tag	HCWs (shoes)		Commercial (MediHandTrace)		Patient area entry/exit events	
		RFID reader	Floor (embedded under the dispenser, patient room, and area entrance)		Commercial (MediHandTrace)		Patient area entry/exit events	
		Dispenser	Patient room and hallway		Commercial (MediHandTrace)		Patient area entry/exit events	
	**Jain et al** **[83]**							
		RFID tag	HCWs (tag)		Research		Patient room entry/exit events	
		RFID reader	Dispenser and patient room entrance		Research		Patient room entry/exit events	
		Dispenser	Patient room and hallway		Research		Patient room entry/exit events	
	**Johnson et al** **[84]**							
		RFID tag	HCWs (tag)		Research		Patient room entry/exit events	
		RFID reader	Patient room entrance		Research		Patient room entry/exit events	
		Dispenser	Patient room entrance		Research		Patient room entry/exit events	
	**Radhakrishna et al** **[99]**							
		RFID tag	HCWs (tag)		Research		Patient area entry/exit events	
		RFID reader	Patient trolley (around patient bed)		Research		Patient area entry/exit events	
		Dispenser	Patient trolley (around patient bed)		Research		Patient area entry/exit events	
		Motion sensor	Patient trolley (around patient bed)		Research		Patient area entry/exit events	
	**Levin et al** **[90]**							
		RFID tag	HCWs (bracelet)		Research		Patient area entry/exit events	
		RFID reader	Patient bed, Dispenser		Research		Patient area entry/exit events	
		Dispenser	N/A^b^		Research		Patient area entry/exit events	
		IMU^c^	HCWs (bracelet)		Research		Patient area entry/exit events	
	**Pleteršek** **et al** **[97]**							
		RFID tag	HCWs (tag)		Research		Hand hygiene events	
		Ethanol sensor	HCWs (tag), Patient room entrance		Research		Hand hygiene events	
**Infrared**								
	**Baslyman et al** **[72]**							
		Infrared transmitter	Patient bed, patient room entrance, dispenser, and hallway		Commercial (Ekahau)		Patient area entry/exit events	
		Infrared receiver	HCWs (tag)		Commercial (Ekahau)		Patient area entry/exit events	
		Dispenser	Patient room		Commercial (Ekahau)		Patient area entry/exit events	
	**Boyce et al** **[74]**							
		Infrared transmitter	Patient bed, dispenser, hallway, and nurse station		Research		Patient area entry/exit events	
		Infrared receiver	HCWs (tag)		Research		Patient area entry/exit events	
		Dispenser	N/A		Research		Patient area entry/exit events	
	**Levchenko et al** **[86-89] and Pong et al** **[98]**							
		Infrared transmitter	Individual patient environments, room entrances, shared bathrooms, dirty utility rooms (ceiling), and dispenser		Research		Patient room entry/exit events	
		Infrared receiver	HCWs (tag)		Research		Patient room entry/exit events	
		Wall-mount dispenser	N/A		Research		Patient room entry/exit events	
		Wearable gel dispenser	HCWs		Research		Patient room entry/exit events	
	**Dyson et al** **[77]**							
		Infrared transmitter	Patient room and area entrance and sink (ceiling)		N/A		Patient area entry/exit events	
		Infrared receiver	HCWs (tag)		N/A		Patient area entry/exit events	
		Ethanol sensor	HCWs (tag)		N/A		Patient area entry/exit events	
**Ultrasound**								
	**Fisher et al** **[79]**							
		Ultrasound transmitter	Patient bed and dispenser		N/A		Patient area entry/exit events	
		Ultrasound receiver	HCWs (tag)		N/A		Patient area entry/exit events	
		Dispenser	Patient room		N/A		Patient area entry/exit events	
	**Srigley et al** **[100]**							
		Ultrasound transmitter	HCWs (tag)		N/A		Hand hygiene events	
		Ultrasound receiver	Patient room, hallway, and dispenser		N/A		Hand hygiene events	
		Dispenser	N/A		N/A		Hand hygiene events	
**Bluetooth low energy (BLE)**								
	**Karimpour et al** **[85]**							
		BLE transmitter	Room		Research		Patient area entry/exit events	
		BLE receiver	HCWs (smartphone)		Research		Patient area entry/exit events	
	**Misra et al** **[94]**							
		BLE transmitter	Patient bed and dispenser		Research		Patient area entry/exit events	
		BLE receiver	HCWs (smartphone)		Research		Patient area entry/exit events	
		Dispenser	Patient bed		Research		Patient area entry/exit events	
	**Marques et al** **[91]**							
		BLE transmitter	Patient room and area entrance, sink, and dispenser		Research		Patient area entry/exit events	
		BLE receiver	HCWs (smartphone)		Research		Patient area entry/exit events	
		Dispenser	Patient room and area entrance		Research		Patient area entry/exit events	
**IEEE 802.15.4/ZigBee**								
	**Marra et al** **[67] and Filho et al** **[78]**							
		ZigBee transmitter	HCWs (tag)		Commercial (Infectrack System)		Patient area entry/exit events	
		ZigBee receiver	Patient bed and dispenser		Commercial (Infectrack System)		Patient area entry/exit events	
		Dispenser	Patient room		Commercial (Infectrack System)		Patient area entry/exit events	
	**Fries et al** **[80], Herman et al** **[81], Hornbeck et al** **[82], Polgreen et al** **[92], and Monsalve et al** **[95,96]**							
		IEEE 802.15.4 transmitter	Patient bed and dispenser		Research		Patient area entry/exit events	
		IEEE 802.15.4 receiver	HCWs (tag)		Research		Patient area entry/exit events	
		Dispenser	Patient room		Research		Patient area entry/exit events	
**Wi-Fi**								
	**Wan et al** **[101]**							
		Wi-Fi transmitter	Room and sink		Research		Hand hygiene events	
		Wi-Fi receiver	HCWs (tag)		Research		Hand hygiene events	
		Sink	Room		Research		Hand hygiene events	

^a^HCW: health care worker.

^b^N/A: not applicable.

Of the 32 studies, 3 (9%) studies developed RTLSs based on BLE technology [85,91,94]. BLE or Bluetooth is a wireless technology standard used for exchanging data between devices through ultra–high-frequency radio waves. These BLE-based RTLSs also contained transmitters (or beacons), BLE receivers, and dispensers. These transmitters were used as location reference points by placing BLE transmitters in health care settings. BLE receivers brought by HCWs could detect HCWs’ real-time location to infer patient room entry/exit events. Unlike the aforementioned RTLSs, BLE receivers could be HCWs’ own smartphones instead of carrying additional equipment. To measure hand hygiene events, dispensers triggered the embedded BLE transmitters once they were actuated.

Of the 32 studies, 8 (25%) studies used IEEE 802.15.4 or ZigBee-based RTLSs [67,78,80-82,92,95,96]. IEEE 802.15.4 is a wireless standard capable of low-power, low-cost wireless communication between devices with lower power consumption. ZigBee is a wireless mesh network specification based on the IEEE 802.15.4 standard [118]. Similar to other RTLSs, they comprise transmitters, receivers, and dispensers. Transmitters were either carried by HCWs or placed in a health care environment. Two individual systems were used in the studies, including one commercial system (Infectrack System, i-HealthSys) based on ZigBee and one in-house system based on IEEE 802.15.4. After collecting the relative distance and/or HCWs’ identity from transmitters, receivers could identify HCWs’ movement when HCWs entered or exited patient areas. The transmitters or receivers were also embedded inside dispensers to recognize hand hygiene events.

The last technology used in RTLSs was Wi-Fi [101]. Wi-Fi is a family of wireless network protocols for building wireless network connections between devices through radio waves. Wi-Fi transmitters were deployed across a room and above a sink, and when HCWs triggered the dispenser next to the sink, the dispenser transmitted the dispensing event to a server through the sink transmitter. The receivers were carried by HCWs, scanned for transmitters in the environment, and periodically uploaded their location to a server.

### Quality Monitoring Systems

Of the 89 studies, 21 (24%) studies evaluated hand hygiene quality as performed by HCWs, grouped into 2 categories based on their measurement methods: (1) compliance with the WHO 6-step hand hygiene techniques (14/21, 67%) and (2) surface coverage or illumination reduction of fluorescent substances (7/21, 33%).

#### Compliance With WHO 6-Step Hand Hygiene Techniques

Of the 21 studies, 14 (67%) studies used a variety of sensors to monitor hand hygiene quality based on compliance with the WHO 6-step hand hygiene techniques (Figure 2). A common practice was to detect the duration of hand hygiene, which is considered a key indicator of quality [13,119]. Furthermore, these systems could recognize HCWs’ hand motions as belonging to the individual steps from the WHO 6-step hand hygiene techniques. As such, these systems provided more details regarding HCWs’ hand hygiene performance, including missed steps and out-of-order sequences, as noncompliance with all steps of hand hygiene procedures fails to cover all skin surfaces [14,20]. In these studies, sensors were either placed in the environment or attached to HCWs to monitor their hand hygiene performance (details are included in Table 5).

**Table 5:** Description of studies monitoring compliance with the WHOa 6-step hand hygiene techniques.

Paper and system description				Device location		System type		System metrics (compliance with hand hygiene techniques)
**Environmental sensor**								
	**Khan et al** **[106]**							
		RGB^b^ camera	Sink		Research		Hand hygiene duration	
		Motion sensor	Sink		Research		Hand hygiene duration	
	**Lacey et al** **[103]**							
		RGB camera	Sink		Commercial (SureWash)		An unknown number of individual steps (WHO 6-step hand hygiene technique)	
	**Camilus et al** **[102]**							
		Depth camera	Sink		Research		6 individual steps (WHO 6-step hand hygiene technique) and 1 wild hand hygiene technique	
	**Zhong et al** **[104]**							
		Depth camera	Sink		Research		9 individual steps (WHO 6-step hand hygiene technique)	
	**Khamis et al** **[105]**							
		mmWave radar	Sink		Research		9 individual steps (WHO 6-step hand hygiene technique)	
**Wearable sensor**								
	**Galluzzi et al** **[52,68]**							
		IMU^c^	HCWs^d^ (wristwatch)		Research		12 individual steps (WHO 6-step hand hygiene technique), 1 wild hand hygiene technique	
	**Li et al** **[69]**							
		IMU	HCWs (wristwatch)		Research		13 individual steps (WHO 6-step hand hygiene technique)	
	**Wijayasingha et al** **[70]**							
		IMU	HCWs (wristwatch)		Research		9 individual steps (WHO 6-step hand hygiene technique)	
		Microphone	HCWs (wristwatch)		Research		9 individual steps (WHO 6-step hand hygiene technique)	
	**Banerjee et al** **[107]**							
		IMU	HCWs (armband)		Research		6 individual steps (self-defined hand hygiene technique)	
	**Kutafina et al** **[108,109]**							
		IMU	HCWs (armband)		Research		9 individual steps (WHO 6-step hand hygiene technique)	
		sEMG^e^	HCWs (armband)		Research		9 individual steps (WHO 6-step hand hygiene technique)	
	**Wang et al** **[110]**							
		IMU	HCWs (armband)		Research		14 individual steps (WHO 6-step hand hygiene technique)	
		sEMG	HCWs (armband)		Research		14 individual steps (WHO 6-step hand hygiene technique)	
	**Zhong et al** **[41]**							
		RGB camera	HCWs (chest)		Research		7 individual steps (self-defined hand hygiene technique)	

^a^WHO: World Health Organization.

^b^RGB: red green blue.

^c^IMU: inertial measurement unit.

^d^HCW: health care worker.

^e^sEMG: surface electromyography.

Of the 14 studies, 5 (36%) studies measured compliance with the WHO 6-step hand hygiene techniques by placing sensors in the environment [102-106]. Khan et al [106] placed an RGB camera and a motion sensor above the sink in operation rooms to monitor HCWs’ hand hygiene duration. Lacey et al [103] used a commercial automatic video auditing system (SureWash, GLANTA Ltd) to monitor HCWs’ compliance with the WHO 6-step techniques. Camilus et al [102] and Zhong et al [104] installed an RGB-depth camera (Kinect) above a sink to record hand hygiene events. Hand hygiene videos with stereo information were then analyzed by classifying each frame as an individual step from the 6-step hand hygiene techniques. Instead of using optical sensors, Khanmis et al [105] installed an mmWave sensor above a sink to measure hand hygiene performance. The mmWave is a sensing technology for detecting objects and provides the range, velocity, and angle of these objects. By using the generated frames from mmWave signals, they could classify each frame as one of the nine individual steps in line with the 6-step hand hygiene techniques.

Of the 14 studies, 9 (64%) studies monitored compliance with hand hygiene guidelines by attaching wearable sensors to HCWs [41,52,68-70,107-110]. Of these, the IMU was the most popular sensor and was used in 89% (8/9) of studies with several supplementary sensors. As mentioned above, the IMU can measure a body’s specific force, angular rate, and orientation. Approximately 44% (4/9) of studies used the IMU of wristwatches to collect physical signals during hand hygiene events and classified hand motion within a certain time frame as one of the several individual steps of the 6-step hand hygiene techniques [52,68-70]. In addition, microphones have been combined with IMUs to evaluate hand hygiene performance, as the additional audio data could further improve the system accuracy [70]. Owing to hygiene reasons, 44% (4/9) of studies used sensor armbands (Myo armband, North Inc) with IMU to detect HCWs’ compliance with hand hygiene techniques [107-110]. Of these 4 studies, 3 (75%) studies used both IMU and surface electromyography (sEMG) sensors from Myo armbands to recognize individual steps in line with 6-step hand hygiene techniques [108-110]. The sEMG sensor is an electrochemical sensor that detects biopotentials using electrodes placed on the skin. In contrast to the aforementioned studies, Zhong et al [41] attached an RGB camera to HCWs’ chests. The camera recorded HCWs’ hand hygiene practices, and then the collected RGB videos were processed by a deep learning algorithm (two-stream convolutional neural network) to classify hand motions into 7 self-defined hand hygiene steps.

#### Surface Coverage or Illumination Reduction of Fluorescent Substances

Of the 21 studies, 7 (33%) studies used fluorescent substances to automatically examine hand hygiene quality by computer vision algorithms. However, the means of detecting the quality of handwash and hand rub were distinct. For handwash, participants first applied fluorescent dye on their entire hands and then washed their hands with soap and tap water thoroughly. For hand rub, a hand disinfectant was mixed with a fluorescent dye, and participants used the disinfectant to perform an episode of hand rub. Then, their hands were checked under a UV light lamp and photographed using RGB cameras for further analysis. By comparing the disinfected areas that glowed under UV light and were free from pathogens, Lehotsky et al [120] stated that fluorescent substances could highlight the areas of the hand surface that were adequately disinfected with acceptable accuracy (95% sensitivity and 98% specificity). UV tests have been widely used to assess hand hygiene quality in medical education because of their easy application, low associated costs, and well-visible results [22].

There were two main ways to automatically analyze the collected RGB images: detecting illumination reduction before and after an episode of handwash or measuring the surface coverage of fluorescent substances (details included in Table 6). Approximately 29% (2/7) of studies calculated the illumination difference of fluorescent substances before and after an episode of handwash using Adobe Photoshop (Adobe Inc) and MATLAB (The Math Works, Inc) [25,111]. Hand hygiene quality was then measured by the value of illumination difference, where a bigger difference indicates better hand hygiene performance and vice versa.

Of the 7 studies, 5 (71%) studies analyzed the collected images from both handwash and hand rub by measuring the surface coverage of fluorescent substances [112-116]. For hand rub, the hand rub quality was acceptable if all areas were bright without dark spots, therefore suggesting that all parts of the hand were covered homogeneously with disinfectant [22]. Approximately 40% (2/5) of studies focused on measuring the surface coverage of fluorescent substances after hand rub by applying clustering algorithms [112,113]. For handwash, as fluorescent substances contaminated hands in advance, the handwash quality was measured by the range of cleaned hand areas (dark areas). Approximately 60% (3/5) of studies applied specific threshold values or deep learning algorithms to measure handwash quality [114-116].

**Table 6:** Description of studies monitoring surface coverage or illumination reduction of fluorescent substances.

Paper and system description				Device location		System type		System metrics (illumination reduction or surface coverage)
**Illumination reduction**								
	**Deochand et al** **[25]**							
		Fluorescent substance	HCWs^a^ (hand)		Research		Illumination reduction (whole hand)	
		UV lamp	Opaque box		Research		Illumination reduction (whole hand)	
		RGB^b^ camera	Opaque box		Research		Illumination reduction (whole hand)	
	**Pellegrino et al** **[111]**							
		Fluorescent substance	HCWs (hand)		Research		Illumination reduction (whole hand)	
		UV lamp	Dark room		Research		Illumination reduction (whole hand)	
		RGB camera	Dark room		Research		Illumination reduction (whole hand)	
**Surface coverage**								
	**Srisomboon et al** **[112]**							
		Fluorescent substance	HCWs (hand)		Research		Surface coverage (pixel)	
		UV lamp	Opaque box		Research		Surface coverage (pixel)	
		RGB camera	Opaque box		Research		Surface coverage (pixel)	
	**Szilágyi et al** **[113]**							
		Fluorescent substance	HCWs (hand)		Research		Surface coverage (pixel)	
		UV lamp	Opaque box		Research		Surface coverage (pixel)	
		RGB camera	Opaque box		Research		Surface coverage (pixel)	
	**Yamamoto et al** **[114,115]**							
		Fluorescent substance	HCWs (hand)		Research		Surface coverage (segment)	
		UV lamp	Opaque box		Research		Surface coverage (segment)	
		RGB camera	Opaque box		Research		Surface coverage (segment)	
	**Naim et al** **[116]**							
		Fluorescent substance	HCWs (hand)		Research		Surface coverage (pixel)	
		UV lamp	Opaque box		Research		Surface coverage (pixel)	
		RGB camera	Opaque box		Research		Surface coverage (pixel)	

^a^HCW: health care worker.

^b^RGB: red green blue.

## Discussion

Recently, there has been increased interest in developing electronic monitoring systems to serve as an alternative or supplementary hand hygiene monitoring approach [19]. However, electronic hand hygiene monitoring systems do have limitations. The following sections discuss the limitations related to accuracy, data integration, privacy and confidentiality, potential risks, usability, associated costs, and infrastructure improvements [19,121].

### System Accuracy

The system accuracy of electronic hand hygiene monitoring systems is the top concern for HCWs [121,122]. However, systems come with different metrics without standardized measurement tools. System accuracy is also affected by technical issues and geometric constraints.

The metrics often vary substantially in different types of electronic hand hygiene monitoring systems. For electronic hand hygiene compliance monitoring systems, the metrics are based on the number of detectable moments for hand hygiene described by the WHO (Figure 1). A total of 4 different metrics were mentioned in the included studies: (1) hand hygiene events, (2) patient room entry/exit events, (3) patient area entry/exit events, and (4) the WHO 5 moments for hand hygiene. Similarly, the metrics for electronic hand hygiene quality monitoring systems are also disparate. One way to measure HCWs’ hand hygiene quality is through detecting their compliance with the WHO 6-step hand hygiene techniques (Figure 2). However, different systems often recognize different sets of individual steps of standardized techniques, which can vary between 6 and 14 individual steps. Detecting illumination reduction or surface coverage of fluorescent substances is another way to measure hand hygiene quality; however, different studies come with different metrics. Several systems can detect pixel or segment levels of fluorescent areas from the collected RGB images; however, others measure the illumination reduction of the entire hand. Therefore, system results may not accurately reflect HCWs’ hand hygiene compliance and quality, and results cannot be compared between different studies without further processing.

Technical issues dramatically affect system accuracy. One of the major concerns is hardware limitations, which result in systems not functioning well under certain situations. For instance, infrared-based RTLS could fail to work if an infrared transmitter or receiver taken by a person is obscured by objects or cloths as the infrared wave cannot penetrate opaque materials [123]. Systems using ethanol sensors to track alcohol-based hand rubs cannot sense HCWs’ handwash events [77]. Systems solely relying on motion sensors (ie, without user identity) cannot provide information on who enters or exits patient rooms. Other systems also suffer from reflected signals, signal noise, and interference. Moreover, the algorithms used in these systems may introduce a variety of errors. An example is that machine learning algorithms used to recognize HCWs’ compliance with WHO 6-step hand hygiene techniques can generate incorrect classifications [110]. In some extreme cases, these algorithms may not correctly recognize any individual steps and provide an entire sequence of erroneous predictions. Thus, both hardware and algorithm limitations need to be considered when implementing hand hygiene monitoring systems, and effective validation of an electronic hand hygiene monitoring system is required to identify associated technical issues.

System accuracy is also influenced by geometric constraints. To protect patient privacy, studies may attach a curtain in front of cameras [36] or point them toward nonsensitive regions only (handwashing sinks and sanitizer dispensers) [38], which may not allow observation of all hand hygiene opportunities and events and further affect system accuracy. Furthermore, systems based on wearable devices are restricted by device position. For example, recent studies have relied on sensor armbands to detect hand hygiene quality; however, their system accuracy is greatly affected by the actual armband position on the arm [110].

### Data Integration

The use of multiple types of sensor data and system records raises new challenges for data integration. Systems use multiple sensors to collect more reliable, accurate, and useful information required for hand hygiene monitoring; however, sensor data fusion comes with problems and issues. One of the most common issues is sensor registration and calibration, as individual sensors have their own local reference frames [124]. Studies applied varying technologies to convert different data from multiple sensors (eg, IMU and sEMG) into one reference frame and starting time, including network time protocols, event-based synchronization methods, and their combination [125,126]. During data fusion and calibration, diverse formats of sensor data could also generate noise and ambiguity, resulting in competitive and conflicting errors, and adding redundancy of sensor data is one of the solutions to increase system reliability [124]. Other issues with multiple sensor data include granularity, timescale, and frequency [124].

Integrating hand hygiene data observed by different systems is another challenge. To increase result accuracy and credibility, studies might use multiple complementary systems to monitor hand hygiene compliance or quality among the same group of HCWs. However, the metric for each observation method was different, and a lack of correlation with their results raised concerns regarding data validity [32]. In addition, different data and result formats raise issues of data integration and require conversion. Moreover, systems could simultaneously observe hand hygiene compliance and quality; however, the means to store and retrieve the records of compliance rate and quality are unclear [41,52,68-70].

### Privacy and Confidentiality

Privacy and confidentiality are two other major concerns associated with electronic hand hygiene monitoring systems. Privacy concerns are known to influence HCWs’ attitudes toward electronic hand hygiene monitoring systems [19]. Some HCWs perceive these systems as an invasion of their privacy and a pretext for constant surveillance of their daily activities, which makes HCWs distrust these systems and refuse to change their hand hygiene behaviors [121]. Electronic hand hygiene monitoring systems also create special challenges regarding patient privacy [127]. Studies using video cameras to monitor all 5 moments of hand hygiene would require constant video surveillance of patients and patient rooms, resulting in violation of patient privacy [26]. However, limited studies have mentioned patient privacy protection before implementing electronic hand hygiene systems. Moreover, constant surveillance through electronic hand hygiene monitoring systems might raise legal issues, resulting in systems that are unpractical in health care settings, especially when involving cameras and microphones.

The continuous collection of personal data in unprecedented volumes also raises data security concerns [128]. During data collection and storage, users’ personal information can be exposed to unauthorized third parties, and the collected data can also be modified or altered through communication protocols (eg, Wi-Fi and Bluetooth) [128]. Furthermore, use scenarios of the collected data are another noticeable concern in hand hygiene monitoring systems for HCWs. Ellingson et al [122] noted that HCWs were worried about the potential use of adherence data for punitive purposes. Thus, an efficient communication mechanism should be established to provide information to HCWs on what data will be collected and stored and how data will be used [121].

### Potential Risks

HCWs may face some potential risks caused by electronic hand hygiene monitoring systems. One potential risk is UV-related skin and eye damage caused by UV lamps, which are used to detect HCWs’ hand hygiene quality [129,130]. Efficient preventive measures should be placed to protect HCWs’ safety and control their daily exposure under a threshold limit of 3.0 mJ/cm^2^ [129]. Wearable sensors have gained popularity to assess HCWs’ hand hygiene quality, especially wristwatches. However, wearing rings, wristwatches, and bracelets could cause hand contamination [131]; therefore, it is challenging to use wristwatches to monitor hand hygiene procedure compliance, as it can possibly defeat the purpose. Moreover, Ward et al [26] noted that during the demolition and installation of monitoring systems in health care, the released particulates such as mold or fungus might increase the risk of infection.

Another risk of deploying electronic hand hygiene systems is radio-frequency interference (RFI) with medical devices. RFI, known as a subset of electromagnetic interference, has been reported to cause medical device failure because of interference from various emitters of radio-frequency energy [132]. Badizadegan et al [133] reported that RFI could also result in erroneous laboratory results. Specifically, van der Togt et al [134] noted that RFID might induce potentially hazardous incidents in medical devices. To prevent RFI-associated medical device failures, system designers and device manufacturers should ensure conformance with current RFI standards, and on-site electromagnetic interference tests are required during implementation [132].

### Usability

Another challenge for implementing electronic hand hygiene monitoring systems in health care is usability, as the technology may interrupt HCWs’ daily workflow to ensure the proper functioning of systems. These usability problems consist of hardware and information delivery. Conway et al [121] summarized hardware-associated usability problems of compliance monitoring systems, including wearable tags (1) as heavy, bulky, and difficult to use; (2) requiring battery power, but batteries are not durable with frequent battery failures; and (3) requiring HCWs to wear them in certain positions. Other usability problems, such as limited sensing range and angles, require HCWs to change their behavior to ensure that systems work properly [77].

Similarly, usability issues also exist when delivering HCWs’ hand hygiene performance information. For hand hygiene compliance monitoring, systems use different types of instant prompts (eg, visual reminders, auditory reminders, vibrations, face-to-face feedback, and olfactory stimulus) to remind HCWs regarding missed hand hygiene opportunities; however, these prompts are associated with several usability problems. For example, Dyson et al [77] noted that systems using visual prompts with a red light could cause patient anxiety. Regarding instant prompts for inadequate hand hygiene quality, most systems are designed for medical training purposes, and thus, efficient delivery of instant feedback to HCWs about hand hygiene quality and integrating these systems into their daily routines are still open challenges.

### Associated Costs and Infrastructure Improvements

Implementing an electronic hand hygiene monitoring system in health care facilities comes with high costs and infrastructure improvements [19,26,121]. Using electronic systems first requires expenditure on equipment and installation costs, which vary with the selected systems [19,26,121]. Morgan et al [59] estimated that the installation of electronic dispenser–assisted systems in a 15-bed intensive care unit requires a cost between US $30,000 and US $40,000. Another study installed 21 video cameras in the hallways and patient rooms of a 17-bed intensive care unit, costing US $50,000 [38]. For community settings, installing a complete set of electronic hand hygiene monitoring systems is not realistic. Instead of fixing sensors in the environment, studies attached wearable sensors to HCWs or embedded sensors into soap bars to track HCWs’ hand hygiene events from their daily routines, which are more scalable and economical.

Except for expenditures on equipment and installation costs, maintenance and personnel costs represent a larger part of system-associated costs. Maintenance costs include system updates, hand rub and soap supplies, an increase in monitored HCWs, and replacement of batteries and defective devices [19]. For in-house systems, technology does not guarantee accurate measurements and requires continuous iteration developments, resulting in maintenance costs and increased personnel needs. Application-assisted direct observation and camera-assisted observation with human auditors are associated with high personnel costs, as these systems require in-house or remote auditors to continually observe hand hygiene opportunities and events.

The installation of electronic hand hygiene systems may disrupt physical infrastructure and require infrastructure improvements. Conway et al [121] noted that infrastructure improvements comprise existing dispenser replacement and fixed hard wiring. As wireless network infrastructure also dramatically affects the system performance, it should be arranged and updated when deploying such systems in health care facilities with outdated network infrastructure.

### Performance Feedback

An important but sometimes overlooked aim of deploying electronic hand hygiene monitoring systems in health care settings is to provide educational interventions to HCWs and improve their practices. The intervention methods used in the included studies comprised instant prompts and periodic summaries.

To remind HCWs about missed hand hygiene opportunities, systems may provide instant prompts when noncompliance is detected. Instant prompts comprise visual reminders, auditory reminders, vibrations, face-to-face feedback, olfactory stimuli, and their combinations. To improve HCWs’ hand hygiene quality, systems also provide instant prompts when detecting hand hygiene events with inadequate quality. Instant prompts include reminding HCWs about missed steps and disordered sequences of the WHO 6-step hand hygiene techniques and visualizing unclean areas from recorded UV test images. Periodic summaries are also widely adopted to improve HCWs’ hand hygiene compliance and quality. Systems deliver periodic summaries to HCWs through reports, dashboards, games, notice boards/monitors, face-to-face feedback, and their combinations.

The included studies also delivered hand hygiene feedback by combining both instant prompts and periodic summaries. For example, Ellison et al [51] adopted auditory reminders as instant prompts and delivered periodic summaries through specific monitor screen savers to remind HCWs of hand hygiene compliance.

Nevertheless, each instant prompt type is associated with specific drawbacks. For visual reminders, Dyson et al [77] noted that red light light-emitting diodes (LEDs) on badges might cause patient anxiety, so the color of badge LEDs should be adjustable and provide an option to disable the LEDs when necessary. Regarding auditory reminders, Baslyman et al [72] noted that sending audible alerts during the night is not acceptable as most patients are sleeping. Face-to-face feedback is associated with the Hawthorne effect, which causes different hand hygiene behaviors from their daily routines [30]. Using unpleasant odors is also not suitable for most health care facilities as they may cause physical discomfort.

Regarding periodic summaries, designing understandable periodic summaries for HCWs with different educational backgrounds is a challenge [121]. Conway et al [48] noted that HCWs or managers might have difficulty reading and interpreting periodic reports with charts. Efficiently disseminating collected information to HCWs and keeping them informed is challenging as well, as many HCWs have reported never or inconsistently receiving their performance information [48]. Moreover, ensuring that periodic summaries are used to drive hand hygiene improvement instead of punishment is another challenge. Hand hygiene improvement might be short-lived and moderate without HCWs’ engagement, constant feedback delivery, detailed action plans, and leadership support [121].

By constantly delivering feedback to HCWs and educating HCWs and medical students on the importance of hand hygiene and the correct procedures, HCWs are likely to improve their hand hygiene techniques and habits. In Multimedia Appendices 3 [53,66,67,77,79,89,98,111], 4 [35,38,39,44,48,51,79,106], and 5 [30,51,88,89], we summarize the performance improvements of HCWs in studies that implemented instant prompts, periodic summaries, or their combinations. However, HCWs have diverse feedback needs. For example, Conway et al [121] and Levchenko et al [89] noted that most HCWs prefer instant prompts rather than periodic summaries, and their compliance rates increased immediately after receiving instant prompts. Nevertheless, Levchenko et al [89] also mentioned that a few HCWs improved their compliance only after they reviewed their individual results.

### Implications

Owing to the high level of heterogeneity of the included studies, it is difficult to compare and analyze data across studies. A noticeable difference across the included studies was the variety of system metrics. To generate quantitative analyses, a high degree of standardization is required. Thus, standardized metrics across different hand hygiene monitoring systems need to be established based on system hardware limitations and WHO recommendations. For instance, the number of individual steps of the WHO 6-step hand hygiene techniques can be set to 9 in line with the WHO guideline as steps 3, 6, and 7 (shown in Figure 2) require repeats for both hands.

Given the recent advancements in sensing technologies, hand hygiene monitoring systems can adopt previously unused technology infrastructure or sensors to monitor HCWs’ hand hygiene performance. For example, the aforementioned systems require a dedicated device being carried by HCWs to trace their indoor locations. Li et al [135] achieved device-free indoor location tracking by using commodity Wi-Fi, which has been installed in most health care facilities. Conversely, hand hygiene monitoring systems can apply new algorithms to improve their system accuracy. For example, previous studies adopted a hidden Markov model to classify the individual steps of 6-step techniques or smooth classification results, which assumes that HCWs will perform hand hygiene procedures according to predefined orders. However, once this assumption is relaxed, the performance of these systems dramatically drops [69]. Instead, classification results smoothed by change point detection algorithms (eg, E.Divisive [136]) might ease the performance decrease.

Hand hygiene monitoring systems and collected data can also be used to solve other hand hygiene–related issues. For example, systems detecting surface coverage of fluorescent substances could be considered as an alternative method to validate the efficacy of newly proposed hand hygiene techniques instead of microbiological tests, as fluorescent substances could highlight the hand surface areas that are adequately disinfected with acceptable accuracy [120]. Similarly, studies have used hand hygiene behavior data to monitor participants’ levels of dementia, Alzheimer disease, and obsessive-compulsive disorder [137,138]. Furthermore, hand hygiene compliance history has been used to simulate the transmission of HAIs in health care settings [139].

### Limitations

This study has several limitations. Some relevant studies may have been missed because of the keywords and databases chosen for the search query. Furthermore, some relevant studies may not have been included if they were not published in English, were outside the specified time frame, or did not provide adequate technical information.

Specifically, we included all types of studies regardless of their maturity, as it helps summarize the latest technological developments in electronic hand hygiene monitoring systems. However, early-stage or preliminary studies or methodology studies may present incomplete data or a lack of results. Owing to the heterogeneity of the studies and sparse metrics, we could not conduct a meta-analysis for the study population, system accuracy, and intervention effectiveness. In addition, because of the significant heterogeneity, we could not evaluate the risk of bias for each study using standardized automation tools and assess the certainty of the included studies.

This review describes different technologies for hand hygiene monitoring. Nevertheless, since we adopted the narrative approach to synthesize the outcomes rather than a meta-analysis, we did not assess the risk of bias because of missing results.

### Conclusions

Our review provides an overview of the latest technological developments in electronic hand hygiene monitoring systems that measure compliance or quality. Systems utilize application-assisted direct observation, camera-assisted observation, sensor-assisted observation, and RTLS to monitor HCWs’ compliance rates. For quality monitoring, systems either measure compliance with the WHO 6-step hand hygiene techniques or detect surface coverage or illumination reduction of fluorescent substances. Despite the technologies used in these systems, we identify system-associated issues and challenges, including system accuracy, data integration, privacy and confidentiality, potential risks, usability, and associated costs and infrastructure improvements. Owing to the narrative approach adopted in these studies, more research is required to establish standardized metrics to measure system performance differences among electronic hand hygiene monitoring systems. With sensing technologies and algorithms continually advancing, more research is needed on their implementation to improve system performance and address other hand hygiene–related issues.

## Appendix

Multimedia Appendix 1 Search strategy for PubMed, IEEE Xplore Digital Library, and ACM Digital Library.

Multimedia Appendix 2 Data extraction form.

Multimedia Appendix 3 Hand hygiene improvements of studies with instant prompts.

Multimedia Appendix 4 Hand hygiene improvements of studies with periodic summaries.

Multimedia Appendix 5 Hand hygiene improvements of studies with instant prompts and periodic summaries.

## Abbreviations

BLE: Bluetooth low energy
HAI: health care–associated infections
HCW: health care worker
IMU: inertial measurement unit
LED: light-emitting diode
PRISMA: Preferred Reporting Items for Systematic Reviews and Meta-Analyses
RFI: radio-frequency interference
RFID: radio-frequency identification
RGB: red green blue
RTLS: real-time locating system
sEMG: surface electromyography
WHO: World Health Organization
